# The Impact of Endpoint Definitions on Predictors of Progression in Active Surveillance for Early Prostate Cancer

**DOI:** 10.3390/cancers18020292

**Published:** 2026-01-17

**Authors:** Kieran Sandhu, Artitaya Lophatananon, Vincent J. Gnanapragasam

**Affiliations:** 1 Department of Surgery, University of Cambridge, Cambridge CB2 0QQ, UK; vjg29@cam.ac.uk; 2 Cambridge Prostate Cancer Research and Clinical Trials Group, Cambridge CB2 0QQ, UK; 3 Division of Population Health, Health Services Research and Primary Care Centre, University of Manchester, Manchester M13 9PL, UK; artitaya.lophatananon@manchester.ac.uk; 4 Department of Urology, Cambridge University Hospitals, Cambridge CB2 0QQ, UK

**Keywords:** prostate cancer, active surveillance, STRATCANs, Cambridge prognostic group

## Abstract

**Background/Objectives**: There is conflicting data on which factors predict progression events in active surveillance for early prostate cancer. Here, we explored the value of different clinicopathological variables and whether progression endpoint definitions impact predictive utility. **Methods**: Clinicopathological variables were extracted from the STRATified CANcer Surveillance (STRATCANs) prospective AS database and included biopsy features (core positivity, cancer core length, and percentage core involvement) and MRI features (Likert score, lesion size, and location), as well as baseline PSA density [PSAd] and Cambridge Prognostic Group (CPG). These were tested against AS endpoint definitions of (1) progression to ≥CPG3, (2) any pathological progression and two definitions from the literature, (3) ≥GG3 or change to treatment, and (4) ≥GG4, metastasis or cancer-related mortality. Predictors were assessed using regression analysis. **Results**: Data from 296 men were included (median age, 66; follow-up, 5 years). Progression per definition (1–4) occurred in 46 (15.5%), 54 (18.2%), 84 (28.4%), and 10 (3.4%) men. In univariate analysis using Definition 1, no biopsy parameter was independently predictive of progression, while the MRI Likert score (*p* = 0.02) was the only significant imaging parameter. For Definition 2, core positivity (*p* = 0.003) and MRI Likert score (*p* = 0.01) were significant predictors in univariate analyses, while for Definition 3, tumour core length (*p* = 0.005), core positivity (*p* = 0.002), and MRI Likert score (*p* = 0.003) were all predictive in univariate analyses. In multivariate analysis, however, the only consistent independent predictor was PSAd, regardless of endpoint definition. No variables predicted Definition 4 progression. **Conclusions**: AS endpoint selection appears to define which variables predict progression. Using progression to ≥CPG 3 as an unambiguous AS endpoint, neither biopsy nor MRI variables added incremental value in predicting progression. PSAd, however, appears to be a robust and independent generalisable progression predictor.

## Simple Summary

Active surveillance (AS) is a critically important management strategy for men with favourable-prognosis prostate cancer. However, there is no standardised or internationally agreed-upon definition of a disease progression endpoint for when AS should stop. This has led to uncertainty regarding which baseline variables reliably predict progression. In the literature, there has also been a multitude of proposed biopsy and imaging metrics that are purported to predict AS progression. We utilised the STRATified CANcer Surveillance (STRATCANs) prospective AS database to assess the utility of different clinicopathological variables in predicting progression and against different contemporary AS endpoint definitions. In this study, we found that the AS endpoint definition appears to determine which variables predict progression. Neither the addition of biopsy nor imaging metrics added consistent incremental value in better predicting progression events. PSA density, in contrast, consistently predicted progression events across different endpoint definitions.

## 1. Introduction

Active surveillance (AS) has emerged as one of the most common management options for men with a prostate cancer (PCa) diagnosis, a trend seen both in the UK and globally [1,2,3]. This shift has been driven by the understanding that many early-stage cancers are unlikely to metastasise or cause disease mortality [4,5,6,7]. This widespread adoption has generated significant interest in developing refined AS protocols and defining predictors of disease progression to improve patient follow-up.

However, unlike the standardised protocols for surgery or radiotherapy, no international benchmark or standard exists for what features should be included in AS protocols as predictors of outcome. The long natural history of the disease also means that traditional oncological endpoints, such as metastasis and mortality, are inadequate endpoints for assessing surveillance outcomes. As a consequence, although major guidelines endorse AS for appropriately selected men, there is no international consensus on what defines AS progression, or which specific progression thresholds should prompt treatment [1,2]. Different study groups have adopted and proposed a variety of measurement parameters and endpoints [8,9,10,11,12,13,14,15]. This makes comparisons between protocols and centres highly challenging and has led to a lack of consensus on the optimal endpoint. This is a critical issue because the relevance of outcome predictors is contingent upon the outcome itself being well defined. Consequently, the generalisability of many proposed predictors for AS progression is uncertain, and their relevance may be limited to the specific studies in which they were developed.

The STRATified CANcer Surveillance programme (STRATCANs) is a validated, risk-stratified tool to tailor modern AS based on individual risk (available at https://www.stratcans.com/ accessed on 13 January 2026) [12]. The program’s protocol uses a clearly defined endpoint (progression to ≥Cambridge Prognostic Group 3 [CPG] 3). Tested predictors of this event (namely prostate-specific antigen [PSA] density [PSAd] and starting CPG assignment) have been published and externally validated in large, independent cohorts [16,17]. In STRATCANS, magnetic resonance imaging (MRI) is also an important part of surveillance strategy; however, MRI Prostate Imaging Reporting and Data System (PI-RADS) and Likert score, rather than just visibility, have not added to model discrimination [18]. In contrast, other studies have proposed PI-RADS score or tumour location as associated with progression risk [8,9]. Other studies have emphasised the importance of biopsy metrics such as core positivity or percentage of tumour involvement in biopsy cores [10,11,12,13,14,15]. Much of this data is based on radical prostatectomy histology from patients who might otherwise have been considered suitable for AS, while very few studies have examined the real-world value of these features in natural history studies of men undergoing surveillance.

The current study exploited the comprehensive STRATCANs database, maintained over many years, to explore the additional value of biopsy and MRI imaging features in predicting outcomes, alongside these established factors. Our goal was to see if we could further refine predictors of progression in the STRATCANs model. We also investigated how the predictive power of clinicopathological features might change if different AS endpoints were used.

## 2. Materials and Methods

### 2.1. Cohort Description and Outcomes Reported

Men selecting AS were enrolled into a prospective stratified follow-up program and a continuously audited database (STRATCANs), which we have previously reported (Institutional Approval: Registration Number: PRN 12,505 ID 6505, Approval Date: 23 October 2024) [19]. Briefly, those with disease suitable for STRATCANs include men with CPG1 and CPG2 disease, based on National Institute for Health and Clinical Excellence (NICE) criteria [20]. As previously described, based on CPG and PSAd (PSA level divided by MRI-derived prostate volume), men are allocated to a 3-tier STRATCANs follow-up protocol of increasing surveillance intensity [19]. Details of the CPG tiers are available at https://www.cancerresearchuk.org/about-cancer/prostate-cancer/stages/cambridge-prognostic-group-cpg (accessed on 2 January 2025 ).

### 2.2. Variables Tested for Active Surveillance Progression Endpoints

Clinicopathological variables at baseline were extracted, including age, PSA, PSAd, grade group (GG), CPG at diagnosis, biopsy core positivity rates (cores with cancer divided by cores taken), highest-grade percentage involvement, highest-grade cancer core length (mm), MRI Likert score, lesion size on MRI (width multiplied by length in mm at the maximum dimension expressed as mm^2^), lesion laterality on MRI (right vs. left side of prostate), and lesion location. Likert scores were categorised as MRI invisible (Likert 1–2) and MRI visible lesions (Likert 3–5). MRI lesion location was classified using a pre-defined 27-sector prostate zonal map, allocating each lesion to apex, mid-gland, or base and to anterior, posterior, or lateral sectors within each lobe, with sector codes then applied as per the reference scheme in Supplementary Table S1. The biopsy method at our institution, which is used for the STRATCANs cohort, has been previously extensively detailed [16,18,19]. In brief, all men were investigated by multiparametric MRI prior to biopsy, which was used to guide biopsies (sectoral and targeted) only if there were no lesions. All men underwent transperineal image-guided prostate biopsies.

### 2.3. Outcome Measures

The predictive ability of different variables was tested for the primary AS endpoint outcome in STRATCANs of progression to ≥CPG3 disease [18]. The secondary endpoint definition was any objective pathological (any increase in GG) or radiological stage (any increase in T-stage) progression. We also tested which variables might predict time-to-progression (TTP) events using these two endpoint definitions. Finally, we tested variables for predicting two alternative endpoint definitions used by recent publications on AS: (i) Definition 3—any grade increase to ≥GG3 disease or any decision to change to treatment and (ii) Definition 4—any grade increase to ≥GG4, development of metastases, biochemical recurrence (BCR) after subsequent radical therapy, or PCa-related-mortality [21,22].

### 2.4. Statistical Analyses

Continuous variables were assessed for normality using the Shapiro–Wilk test and summarised as the mean and median with interquartile range. Categorical variables were expressed as frequencies and percentages. PSAd displayed a non-normal distribution, and a log transformation was applied to reduce skew and ensure suitability for regression analysis. A log-transformed PSAd variable was used for all regression analyses. The proportion of available and missing data for each variable is reported in Table 1, Table 2, Table 3 and Table 4 and Supplementary Table S2. To identify predictors of progression, univariate logistic regression was first performed for clinicopathological variables of interest. Results are reported as odds ratios (ORs) and 95% confidence intervals (CIs). Significant variables were then included in a multivariate logistic regression model with outcomes reported as ORs with corresponding 95% CIs. Significant variables from the univariate analysis, as well as baseline CPG and STRATCANs tier, were also included in a Cox proportional-hazards model to determine TTP, with results reported as hazards ratios (HRs) with corresponding 95% CIs reported. TTP was calculated from diagnosis to the first defined progression event (i.e., reaching ≥CPG3 disease or any objective pathological or radiological stage progression). Kaplan–Meier curves and log-rank tests were generated for TTP events stratified by STRATCANs tier. All tests were two-sided, and statistical significance was defined as *p* < 0.05. Statistical analyses were performed using the Stata Statistical Software: Release 18 (StataCorp LLC, College Station, TX, USA) and Jamovi (Version 2.5, Sydney, Australia).

## 3. Results

### 3.1. Cohort Demographics

Data was available for 296 men from the STRATCANs database for this analysis. The median age was 66 years (61–71) (Table 1). Median PSA and PSAd were 6.13 ng/mL (4.47–8.08) and 0.12 ng/mL/mL (0.09–0.17), respectively. At baseline, 206 (73.8%) men had visible MRI lesions (Likert 3–5). Most men had CPG1 and GG1 disease, with 39.2% of men having CPG2 disease and 26.4% with GG2 disease (Table 1). Most entered surveillance under STRATCANs Tier 1 (42.9%; Table 1). The median time on AS for the whole cohort was 4.11 years (2.89–6.53). In total, 150 men remained on AS with a median follow-up of 5.23 years (3.29–8.40). Progression to ≥CPG3 occurred in 46 (15.5%) and, for any pathological/radiological stage progression, in 54 men (18.2%) (Table 1).

### 3.2. Value of Biopsy and MRI Characteristics in Predicting Progression to (CPG3 Disease

The primary endpoint of STRATCANs is progression to ≥CPG3. Using this endpoint definition, there was no statistical association in the univariate analysis between core positivity, cancer core length, and percentage cancer core involvement for progression to ≥CPG3 (*p* = 0.08, *p* = 0.13, and *p* = 0.08, respectively; Table 2). Amongst MRI parameters, only MRI Likert visibility was associated with progression to ≥CPG3 disease (OR 1.34, 95% CI: 1.04–1.72, *p* = 0.02), while MRI lesion size, laterality, or location were not (*p* = 0.56, *p* = 0.97, and *p* = 0.12, respectively; Table 2). However, in a multivariate model, MRI Likert score did not remain an independent predictor (OR 1.32, 95% CI: 0.27–6.51, *p* = 0.73; Supplementary Table S2).

### 3.3. Value of Biopsy and MRI Characteristics in Predicting Any Pathological/Radiological Stage Progression

Using this endpoint, only core positivity amongst biopsy variables showed an association with progression in univariate analyses (OR 9.88, 95% CI: 2.20–44.45, *p* = 0.003) (Table 2). Cancer core length and percentage cancer core involvement showed no association (*p* = 0.12 and *p* = 0.09, respectively). Similar to progression to ≥CPG3 disease, MRI Likert score was predictive (OR 1.28, 95% CI: 1.04–1.56, *p* = 0.01) for any pathological/stage progression in the univariate analysis. However, in a multivariate model, neither core positivity (OR 6.16, 95% CI: 0.70–52.60, *p* = 0.10) nor baseline Likert score (OR 0.70, 95% CI: 0.30–1.60, *p* = 0.39) remained an independent predictor (Supplementary Table S2).

### 3.4. Clinicopathological Characteristics Used to Predict Time-to-Progression

**Figure 1:**
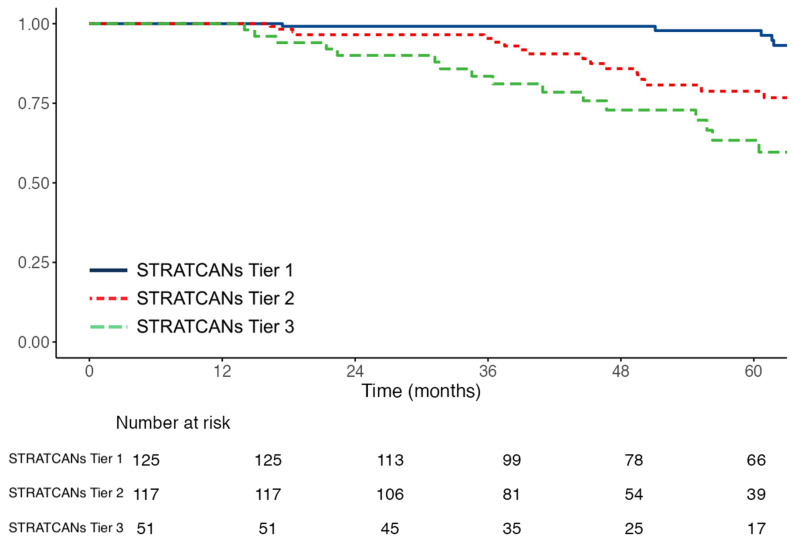
Kaplan–Meier curves showing time to progression to ≥Cambridge Prognostic Group 3 disease stratified by STRATified CANcer Surveillance tier (n = 296). Log—rank: *p* < 0.0001. Numbers at risk at each time point are shown below the *x*-axis.

A key need in AS is the early identification of men who are likely to rapidly progress. Using time to progression for ≥CPG3 disease as the endpoint, PSAd (HR 1.99, 95% CI: 1.41–2.81, *p* < 0.001), MRI Likert score (HR 2.54, 95% CI: 1.17–5.49, *p* = 0.018), and starting AS with CPG2 disease (HR 2.01, 95% CI: 1.28–3.15, *p* = 0.003) were associated with more rapid progression (Table 3). STRATCANs tiering, which includes both PSAd and CPG, therefore, also effectively stratified risk for progression to ≥CPG3 (Table 3, Figure 1). Relative to Tier 1, men starting AS in STRATCANs Tier 2 had an approximate three-fold higher risk of progression to ≥CPG3 (HR 2.51, 95% CI: 1.17–5.41, *p* = 0.02), while men starting AS in STRATCANs Tier 3 had an approximate five-fold higher risk (HR 4.99, 95% CI: 2.28–10.91, *p* < 0.001). Compared to Tier 2, men starting AS in Tier 3 had an approximate two-fold risk of progression to ≥CPG3 disease (HR 1.99, 95% CI: 1.03–3.83, *p* = 0.04). For time to any pathological/radiological stage progression, PSAd (HR 1.83, 95% CI: 1.34–2.48, *p* < 0.001), biopsy core positivity (1.03, 95% CI: 1.02–1.04, *p* < 0.001), and MRI Likert score (HR 2.02, 95% CI: 1.03–3.95, *p* = 0.04) predicted TTP. However, STRATCANs tier upon entering AS did not predict TTP for any pathological progression (Table 3; Supplementary Figure S1).

### 3.5. Progression Endpoint Definition and the Predictive Value of Different AS Variables

Our results suggest that different endpoint definitions of AS progression influence the utility of predictive variables. To further explore this notion, we tested the predictive ability of baseline variables with two other progression endpoints from the recent literature. Progression to Definitions 3 and 4 occurred in 84 (28.4%) and 10 (3.4%) men, respectively. The predictive value of each variable did indeed again vary by endpoint definition (Table 4). Amongst biopsy and MRI characteristics, for Definition 3, only cancer core length (OR 1.13, 95% CI: 1.04–1.22, *p* = 0.005), core positivity (OR 9.72, 95% CI: 2.25–42.10, *p* = 0.002), and Likert score (OR 1.36, 95% CI: 1.12–1.65, *p* = 0.003) were predictive for this endpoint (Table 4). However, in multivariate analysis, neither of these remained predictive (Supplementary Table S2). We did not identify any progression predictors for Definition 4, regardless of the variables analysed (Table 4). PSAd, similar to its value in predicting for ≥CPG3 disease and any pathological progression, was again a univariate variable for the Definition 3 endpoint (OR 3.04, 95% CI: 1.75–5.28, *p* = 0.002) (Table 4). PSAd was also the only one that retained predictive ability when tested in a multivariate analysis for Definition 3 (OR 3.98, 95% CI: 1.26–12.57, *p* = 0.018; Supplementary Table S2). In these multivariate models, only PSAd consistently remained significant.

## 4. Discussion

The principal finding of this study is that the apparent predictors of AS progression vary depending on how progression is defined. Metrics of biopsy and MRI features demonstrate variable associations depending on the chosen endpoint definition. In the context of the STRATCANS framework, which uses baseline PSAd and CPG to stratify progression risk, we were unable to show that any biopsy or MRI metric improved model performance.

Multiple guidelines and AS protocols have proposed biopsy tumour-burden metrics to define eligibility for AS or triggers for intervention [1,2,23]. The current recommendations for which biopsy metrics and what cut-offs to recommend are weak and come mainly from literature reviews of published AS protocols rather than empirical research [24]. The European Association of Urology (EAU) suggests excluding men with GG2 disease and >3 positive cores or ≥50% core involvement in any core, whilst other cohorts have suggested cut-offs of 20% and 33% [2,25,26]. However, the basis of these cut-offs is unclear, with much of the evidence derived from retrospective series of men who had radical prostatectomy [2,14,23,25,26,27]. Where AS series have proposed thresholds, this is conflicting data. In the Canary PASS AS cohort, Newcomb et al. proposed a core positivity cut-off of ≥34% as unsuitable for AS [14]. Conversely, Shee et al. suggested that any core positivity > 10% was associated with AS progression [28]. In this study, we found that not only did biopsy metrics not consistently add value in predicting progression but that the definition of “progression” used affected predictive utility. Taken together, this would suggest that there is little justification for any specific biopsy threshold to be used in either selecting men for AS or in tailoring monitoring.

Prostate lesion visibility and the PI-RAD/Likert scoring system by MRI are integral to modern prostate cancer diagnosis. There is, however, no evidence that these scores affect the outcomes for men who select AS. In this study, MRI Likert score (dichotomised as 1–3 vs. 4–5) was not independently associated with any definition of progression. We also tested for the association of each Likert score categorised separately but similarly found no predictive value. This finding is consistent with other studies that have demonstrated that the PI-RADS score performs poorly as a longitudinal predictor of AS progression [29,30]. Peyrottes et al., for example, also recently reported that PI-RADS 5 lesions were not an adverse feature for men on AS [31]. The findings that no imaging parameter independently predicted progression reinforce that MRI changes alone should not be taken as a justification to move to treatment, consistent with guideline recommendations by the EAU [2,24,32]. Importantly, the absence of incremental predictive value from MRI features should not be interpreted as diminishing the diagnostic utility of MRI in AS.

A key observation from our multivariate analyses is the lack of incremental predictive value provided by biopsy tumour-burden metrics and MRI features once PSAd and baseline GG are considered. PSAd emerged as a consistent clinicopathological variable that independently predicted progression to ≥CPG3 disease, pathological/stage progression, Definition 3 progression, and shorter TTP. This association has also been demonstrated by others [4,5,31,33,34]. In the Toronto AS cohort, Klotz et al. identified PSAd as a strong baseline predictor of AS progression [4]. Similarly, in the John Hopkins programme, Tosoian et al. reported that PSAd > 0.15 was strongly associated with AS progression [5]. Other large AS cohorts, including the PRIAS Göteborg cohort, demonstrated the same relationship between PSAd and AS progression in long-term follow-up [33,34]. This independent predictive value across multiple institutional AS cohorts, combined with its minimal cost and universal availability, makes PSAd a robust and consistent marker for risk-stratified surveillance, and we advocate that it be an integral part of any surveillance practice [35]. In our cohort, TTP analyses demonstrated that baseline CPG also independently predicted earlier progression to ≥CPG3 disease. Both PSAd and baseline CPG are included in the classification method for STRATCANs tiers, and Kaplan–Meier analysis confirmed clear time-dependent separation between tiers, with progressively shorter TTP in higher-tier strata. This is consistent with the findings of Moser et al., who applied the STRATCANs tiers to their series of over 7000 men on AS [36].

Our study raises the important issue of how AS endpoints are selected. Currently, apart from the Irish National Cancer Control Programme AS protocol, there are no guidelines that clearly specify AS endpoints [37]. Inevitably, therefore, there is significant variation between studies. The impact of this can be seen in meta-analyses, where treatment conversion rates vary from 10% to over 50% at 5 years despite nearly identical baseline disease profiles, illustrating the influence of centre-level practice variation [38]. Some studies have defined AS progression using composite endpoints—for example, by combining histological upgrading, radiological change, PSA kinetics, treatment conversion, BCR, or metastases [4,21,22,33]. While these definitions broaden event rates, they also conflate distinct processes. For example, Definition 3 in our study (≥GG3 or conversion to treatment) produced the largest number of univariate significant predictors. However, this definition mixes tumour progression with subjective decisions and thus partially reflects clinician-determined treatment/anxiety thresholds rather than genuine disease progression [21]. Endpoints such as BCR are also questionable for assessing AS outcomes [22]. These events represent subsequent treatment failure rather than surveillance failure. Contemporary protocols would also not keep men on AS long enough to develop events like metastasis or disease-related mortality. These outcomes are of doubtful value in surveillance studies, especially as they are also rare: 1–2% metastasis rates at 10–15 years [4,5,6,7].

This study has several strengths. It draws on a prospectively maintained, real-world AS cohort with consistent MRI and biopsy protocols, ensuring high internal validity and minimising variability in data capture. The inclusion of clinicopathological, biopsy, and MRI variables enabled a comprehensive assessment of potential predictors, and the systematic evaluation of multiple AS progression definitions provided clear insight into how endpoint selection influences predictor behaviour. This represents an important contribution of our work, as it helps explain the variability in reported predictors across the AS literature and underscores the value of adopting clinically meaningful, standardised endpoints. The consistent predictive performance of PSAd across models, together with the strong discriminatory ability of the STRATCANs tiering system, further reinforces the robustness and clinical utility of simple, reproducible metrics. These findings support simple AS protocols that prioritise robust, accessible, and low-cost indicators such as PSAd and baseline CPG over MRI metrics or biopsy features that do not appear to provide incremental value.

Several limitations must also be acknowledged. As a single-centre cohort, the findings may not be fully generalisable to settings with differing biopsy or imaging practices, despite the advantages of protocol stability. Furthermore, our cohort size is small, and so our results do need verifying in future larger series. Progression definitions were applied retrospectively to prospectively collected data, introducing a small risk of misclassification, although this was mitigated by excluding early reclassification events. The low number of advanced progression events (Definition 4) limits our power to evaluate predictors of late-stage outcomes, a common constraint in contemporary AS cohorts. MRI interpretation and biopsy sampling variability, although systematically managed, remain potential sources of measurement noise. Moreover, while STRATCANs has been externally validated, the extended evaluation of MRI and biopsy predictors presented here was not validated in an external dataset and should therefore be interpreted with caution. Finally, the investigation of variable-specific predictive value required complete-case analysis, reducing the analytic sample. Imputation was intentionally avoided, as modelled values could introduce unverifiable assumptions about missingness, distort true variable associations, and diminish the robustness and interpretability of the findings. Taken together, our findings highlight the need for greater standardisation of progression endpoints in AS programs and the use of objective rather than subjective endpoints.

## 5. Conclusions

This study demonstrates that the predictive performance of clinicopathological variables in AS is determined by the progression definition applied. Tumour-burden and MRI metrics show associations with composite, subjective, and objective endpoint definitions but did not persist when progression was limited to a clinically meaningful hard endpoint, i.e., ≥CPG3 progression. These findings underscore the importance of selecting clinically meaningful outcomes when evaluating predictive markers, as heterogeneity in endpoint definition underlies much of the variability seen in AS literature. Harmonisation of progression definitions is necessary. We propose that progression to ≥CPG3 be adopted as a standard outcome in future AS studies and programs. Aligning endpoint definitions around clinically meaningful outcomes could improve comparability across cohorts.

## Figures and Tables

**Table 1:** Baseline clinicopathological features of men enrolled in the AS programme and who met eligibility for analysis in this study. Definition 3: Progression to ≥GG3 or any decision to treat. Definition 4: Progression to ≥GG4, metastasis or prostate cancer-related mortality.

Variable	Value
**Age (years) (n = 296)**	
Mean	66
Median (IQR)	66 (61–71)
**PSA (ng/mL) (n = 296)**	
Mean	6.80
Median (IQR)	6.13 (4.47–8.08)
**PSAd (ng/mL/mL) (n = 293)**	
Mean	0.14
Median (IQR)	0.12 (0.09–0.17)
**MRI Likert score (n = 279)**	**n (%)**
Likert 1–2	73 (26.2)
Likert 3–5	206 (73.8)
**Cambridge Prognostic Group (CPG) (n = 296)**	**n (%)**
CPG 1	180 (60.8)
CPG 2	116 (39.2)
**Grade Group (n = 296)**	**n (%)**
GG1	78 (26.4)
GG2	218 (73.6)
**STRATCANS tier (n = 296)**	**n (%)**
1	127 (42.9)
2	118 (39.9)
3	51 (17.2)
**Years on AS** **whole cohort (n = 296)**	
Mean	4.53
Median (IQR)	4.11 (2.89–6.53)
**Years on AS** **men still on surveillance (n = 150)**	
Mean	5.89
Median (IQR)	5.23 (3.29–8.40)
**Progression event by definition**	**n (%)**
To ≥CPG3	46 (15.5)
Any objective pathological/radiological progression	54 (18.2)
Definition 3	84 (28.4)
Definition 4	10 (3.4)

PSA: Prostate-specific antigen. PSAd: PSA divided by MRI-derived prostate volume. STRATCANs: STRATified CANcer Surveillance tier.

**Table 2:** Univariate analysis of variables tested for association with progression to ≥Cambridge Prognostic Group (CPG) 3 and any pathological/stage progression. Cells in grey are where *p* < 0.05. (* log-transformed).

Variables at Baseline	Progression to ≥CPG 3 Disease Odds Ratio (95% CI) *p*-Value	Any Pathological/Stage Progression Odds Ratio (95% CI) *p*-Value
Age at diagnosis (n = 296)	1.02 (0.98–1.07) *p* = 0.36	1.00 (0.96–1.03) *p* = 0.80
PSA (n = 296)	1.08 (0.99–1.19) *p* = 0.07	0.98 (0.90–1.06) *p* = 0.63
PSA density (PSAd) * (n = 293)	3.64 (1.82–7.28) *p* < 0.001	2.36 (1.36–4.09) *p* = 0.004
Grade Group (n = 296)	1.82 (0.93–3.53) *p* = 0.08	1.07 (0.59–1.95) *p* = 0.82
Cambridge Prognostic Group (n = 296)	2.33 (1.23–4.44) *p* = 0.009	1.12 (0.65–1.92) *p* = 0.68
Core positivity (%) (n = 286)	4.71 (0.85–26.05) *p* = 0.08	9.88 (2.20–44.45) *p* = 0.003
Percentage cancer involvement (%) (n = 146)	1.02 (1.00–1.05) *p* = 0.08	1.02 (1.00–1.04) *p* = 0.09
Cancer core length (mm) (n = 226)	1.08 (0.98–1.19) *p* = 0.13	1.07 (0.98–1.16) *p* = 0.12
MRI Likert score (n = 279)	1.34 (1.04–1.72) *p* = 0.02	1.28 (1.04–1.56) *p* = 0.01
MRI lesion size (mm^2^) (n = 138)	1.00 (1.00–1.01) *p* = 0.56	1.00 (0.99–1.00) *p* = 0.69
MRI lesion laterality (n = 289)	1.00 (0.78–1.30) *p* = 0.97	1.03 (0.83–1.28) *p* = 0.80
MRI lesion location (n = 215)	0.92 (0.82–1.02) *p* = 0.12	0.95 (0.87–1.04) *p* = 0.28

PSA: Prostate-specific antigen. PSAd: PSA divided by MRI-derived prostate volume.

**Table 3:** Cox-proportional hazards modelling of clinicopathologic variables that predict time to progression for ≥Cambridge Prognostic Group (CPG) 3 and any pathological/stage progression. Cells in grey are where *p* < 0.05. * PSAd: PSA divided by MRI-derived prostate volume.

Variable at Baseline	Progression to ≥CPG 3 Disease Hazard Ratio (95% CI) *p*-Value	Any Pathological/Radiological Stage Progression Hazard Ratio (95% CI) *p*-Value
PSA density (PSAd) * (n = 293)	1.99 (1.41–2.81) *p* < 0.001	1.83 (1.34–2.48) *p* < 0.001
Core positivity (%) (n = 286)	-	1.03 (1.02–1.04) *p* < 0.001
Cambridge Prognostic Group (n = 296)	2.01 (1.28–3.15) *p* = 0.003	-
MRI Likert score (n = 279)	2.54 (1.17–5.49) *p* = 0.018	2.02 (1.03–3.95) *p* = 0.04
STRATCANs tier (n = 296)		
STRATCANs Tier 2 vs. Tier 1	2.51 (1.17–5.41) *p* = 0.019	1.52 (0.83–2.77) *p* = 0.17
STRATCANs Tier 3 vs. Tier 1 STRATCANs Tier	4.99 (2.28–10.91) *p* < 0.001	1.63 (0.78–3.40) *p* = 0.20
STRATCANs Tier 3 vs. Tier 2	1.99 (1.03–3.83) *p* = 0.04	1.09 (0.53–2.24) *p* = 0.81

* indicates log-transformed which is in the legend and needs to stay

**Table 4:** Comparative analysis of predictive variables for univariate association with different active surveillance endpoint definitions. Definition 3: Progression to ≥GG3 or any decision to treat. Definition 4: Progression to ≥GG4, metastasis, or prostate cancer-related mortality. Cells in grey are where *p* < 0.05 (* log-transformed). PSA: Prostate-specific antigen. PSAd: PSA divided by MRI-derived prostate volume.

Variable at Baseline	Progression to ≥CPG 3 Disease Odds Ratio (95% CI) *p*-Value	Any Pathological/Stage Progression Odds Ratio (95% CI) *p*-Value	Definition 3 Odds Ratio (95% CI) *p*-Value	Definition 4 Odds Ratio (95% CI) *p*-Value
Age at diagnosis (n = 296)	1.02 (0.98–1.07) *p* = 0.36	1.00 (0.96–1.03) *p* = 0.80	1.00 (0.97–1.04) *p* = 0.94	1.06 (0.96–1.17) *p* = 0.26
PSA (n = 296)	1.08 (0.99–1.19) *p* = 0.07	0.98 (0.90–1.06) *p* = 0.63	1.02 (0.94–1.09) *p* = 0.70	1.05 (0.89–1.25) *p* = 0.56
PSA density (PSAd) * (n = 293)	3.64 (1.82–7.28) *p* < 0.001	2.36 (1.36–4.09) *p* = 0.004	3.04 (1.75–5.28) *p* = 0.002	2.47 (0.68–8.95) *p* = 0.17
Grade Group (n = 296)	1.82 (0.93–3.53) *p* = 0.08	1.07 (0.59–1.95) *p* = 0.82	1.66 (0.95–2.89) *p* = 0.07	1.22 (0.31–4.83) *p* = 0.78
Cambridge Prognostic Group (n = 296)	2.33 (1.23–4.44) *p* = 0.009	1.12 (0.65–1.92) *p* = 0.68	1.48 (0.88–2.48) *p* = 0.14	1.56 (0.44–5.51) *p* = 0.49
Core positivity (%) (n = 286)	4.71 (0.85–26.05) *p* = 0.08	9.88 (2.20–44.45) *p* = 0.003	9.72 (2.25–42.10) *p* = 0.002	0.10 (0.00–12.50) *p* = 0.35
Percentage cancer involvement (%) (n = 146)	1.02 (1.00–1.05) *p* = 0.08	1.02 (1.00–1.04) *p* = 0.09	1.02 (0.99–1.04) *p* = 0.14	0.97 (0.88–1.07) *p* = 0.56
Cancer core length (mm) (n = 226)	1.08 (0.98–1.19) *p* = 0.13	1.07 (0.98–1.16) *p* = 0.12	1.13 (1.04–1.22) *p* = 0.005	0.87 (0.66–1.14) *p* = 0.31
MRI Likert score (n = 279)	1.34 (1.04–1.72) *p* = 0.02	1.28 (1.04–1.56) *p* = 0.01	1.36 (1.12–1.65) *p* = 0.003	0.94 (0.60–1.48) *p* = 0.79
MRI lesion size (mm^2^) (n = 138)	1.00 (1.00–1.01) *p* = 0.56	1.00 (0.99–1.00) *p* = 0.69	1.00 (1.00–1.01) *p* = 0.11	1.00 (0.98–1.01) *p* = 0.83
MRI lesion laterality (n = 289)	1.00 (0.78–1.30) *p* = 0.97	1.03 (0.83–1.28) *p* = 0.80	0.96 (0.78–1.19) *p* = 0.68	1.40 (0.83–2.35) *p* = 0.20
MRI lesion location (n = 215)	0.92 (0.82–1.02) *p* = 0.12	0.95 (0.87–1.04) *p* = 0.28	0.94 (0.86–1.02) *p* = 0.14	1.10 (0.88–1.36) *p* = 0.40

## Data Availability

Data are contained within the article and supplementary materials.

## Supplementary Materials

The following supporting information can be downloaded at https://www.mdpi.com/article/10.3390/cancers18020292/s1, Figure S1: Kaplan-Meier curves showing time-to-progression to any pathological/stage progression stratified by STRATified CANcer Surveillance tier (n = 296). Log—rank: *p* = 0.28. Numbers at risk at each time point are shown below the *x*-axis; Table S1: Allocation method for MRI lesion location. Codes were allocated based on location of the index lesion on MRI (highest Likert score). Zones based on the schema were then allocated a code for statistical analysis. AFMS: Anterior Fibromuscular Stroma. Extensive lesions were any suspicious lesions that covered multiple locations; Table S2: Comparative multivariate analysis of variables tested for association with different active surveillance endpoints. Definition 3: Progression to ≥GG3 or any decision to treat. Cells in grey are where *p* < 0.05. (* log-transformed). PSAd: PSA divided by MRI-derived prostate volume. Definition 4 was not analysed, as no predictors were identified in univariate modelling.

## Abbreviations

The following abbreviations are used in this manuscript:

AS	Active surveillance
PCa	Prostate cancer
CPG	Cambridge Prognostic Group
STRATCANs	STRATified CANcer Surveillance
PSA	Prostate-specific antigen
PSAd	Prostate-specific antigen density
MRI	Magnetic resonance imaging
PI-RADS	Prostate Imaging Reporting and Data System
GG	Grade group
OR	Odds ratio
CI	Confidence interval
TTP	Time to progression
HR	Hazard ratio
BCR	Biochemical recurrence
NICE	National Institute for Health and Care Excellence
EAU	European Association of Urology
