# Supplementary material for: The Impact of Endpoint Definitions on Predictors of Progression in Active Surveillance for Early Prostate Cancer
Source: Cancers (Basel). 2026 Jan 17;18(2):292. doi: 10.3390/cancers18020292 (PMC12839284; doi:10.3390/cancers18020292)
Supplement: Supplementary file 1 [file cancers-18-00292-s001.zip › cancers-4068276-SI.pdf]

Supplementary Table S1. Allocation method for MRI lesion location. Codes were allocated based on location of the index lesion on MRI (highest Likert score). Zones based on the schema were then allocated a code for statistical analysis. *AFMS: Anterior Fibromuscular Stroma. Extensive lesions were any suspicious lesions that covered multiple locations.*

Code for analysis	1	2	3	4	5	6	7	8	9
**Location**	*Right apex*	*Left apex*	*Right mid*	*Left mid*	*Right base*	*Left base*	*AFMS*	*Extensive*	*Not stated*
	5p, 6a/6p, 5a	11p, 12a/12p, 11a	3p, 4a/4p, 3a	9p, 10a/10p, 9a	1p, 2a/2p, 1a	7p, 8a/8p, 7p	15/14/13 as	Multiple zones	Site not stated

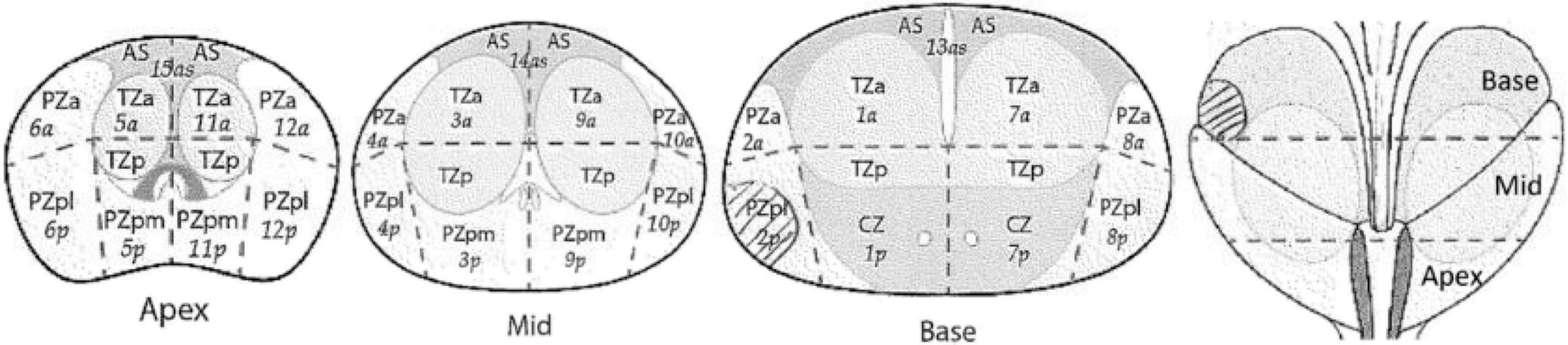

Supplementary Table S2. Comparative multivariate analysis of variables tested for association with different active surveillance endpoints. Definition 3: Progression to ≥GG3 or any decision to treat. Cells in grey are where *p* < 0.05 (21). (* log-transformed). *PSAd: PSA divided by MRI-derived prostate volume. Definition 4 was not analysed, as no predictors were identified in univariate modelling.*

Variable at baseline	Progression to ≥CPG 3 disease<br><br>Odds ratio (95% CI)<br>p-value	Any pathological/stage progression<br><br>Odds ratio (95% CI)<br>p-value	Definition 3<br><br>Odds ratio (95% CI)<br>p-value
**PSA density (PSAd)*(n = 293)**	4.55 (1.32 – 15.64)<br>p = 0.016	2.89 (1.50 – 5.70)<br>p = 0.002	3.98 (1.26 – 12.57)<br>p = 0.018
**Core positivity (%) (n = 286)**	-	6.16 (0.70 – 52.60)<br>p = 0.10	2.37 (0.04 – 144.38)<br>p = 0.68
**Cambridge Prognostic Group (n = 296)**	1.20 (0.38 – 3.84)<br>p = 0.754	-	-
**MRI Likert score (n = 279)**	1.32 (0.27 – 6.51)<br>p = 0.73	0.70 (0.30 – 1.60)<br>p = 0.39	1.08 (0.32 – 3.66)<br>p = 0.90
**Cancer core length (mm) (n = 226)**	-	-	0.75 (0.49 – 1.16)<br>p = 0.20

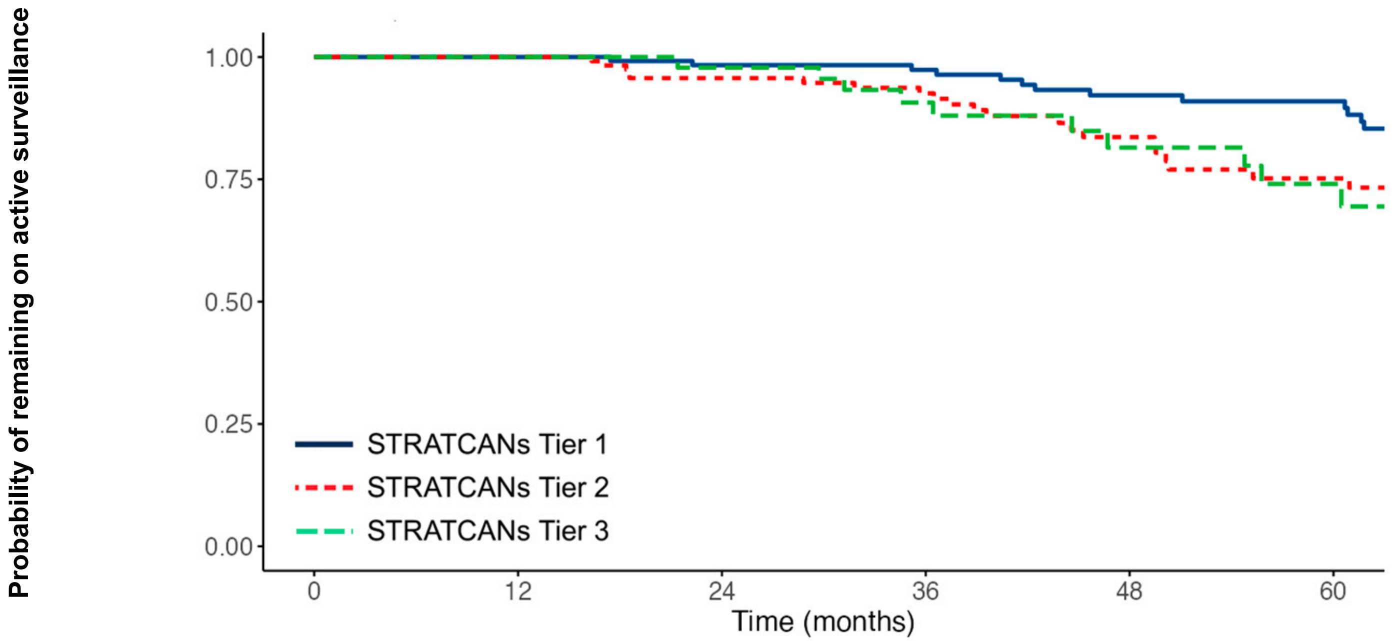

Number at risk

	0	12	24	36	48	60
STRATCANs Tier 1	127	127	115	100	79	67
STRATCANs Tier 2	118	118	107	82	55	40
STRATCANs Tier 3	50	50	44	34	24	16

Supplementary Figure S1. Kaplan-Meier curves showing time-to-progression to any pathological/stage progression stratified by STRATified CANcer Surveillance tier (n = 296). Log—rank: p = 0.28. Numbers at risk at each time point are shown below the x-axis.
